# Spectroscopic details on the molecular structure of pyrimidine‑2‑thiones heterocyclic compounds: computational and antiviral activity against the main protease enzyme of SARS-CoV-2

**DOI:** 10.1186/s13065-022-00881-3

**Published:** 2022-11-02

**Authors:** Doaa S. El Sayed, El-Sayed M. Abdelrehim

**Affiliations:** 1 Chemistry Department, Faculty of Science, Alexandria University, Alexandria, Egypt; 2 Chemistry Department, Faculty of Science, Damanhour University, Damanhur, Egypt

**Keywords:** Computational advance, DFT, Geometrical description, Spectroscopic analyses, Molecular docking

## Abstract

Computational tools in investigating of spectral heterocyclic compounds ranges based on pyrimidine‑2‑thiones, take some importance in identifying their molecular and electronic behavior. Some charcoal heterocyclic compounds were previously synthesized in our laboratory and their experimental results were compared with the computational evaluation. Computational spectroscopic analytical items (IR, NMR and UV–Vis) were calculated using the more popular DFT methods and the predicted results were compared with the reported experimental ones. Quantum and chemical parameters were calculated and molecular electrostatic surface potential (MEP) was studied which predicted the highly electronic sites around the compounds. Some molecular properties (ionization energy, electron affinity, energy gap, hardness, electronegativity, electrophilicity index, static dipole moment and average linear polarizability) of these Schiff bases which were computed at B3LYP/6-31G(d,p) level in aqueous phase. Benchmark analysis was performed for three ab initio functionals such B3LYP, BPV86 and B3PW91 methods to explain the data resulted from NMR spectra. The docking study of some selected previously synthesized compounds was performed using the viral Mpro enzyme protein in compared to a k36 reference ligand inhibitor. The study indicated the ability of the synthesized compounds to form H-bond and hydrophobic (VDW, π-alkyl and π-sulfur) interactions with Mpro enzyme receptor with high inhibition effect of compound L2.

**Supplementary Information:**

The online version contains supplementary material available at 10.1186/s13065-022-00881-3.

## Introduction

Theoretical computational guides highlight an important role in investigating the molecular structure and electronic properties of a wide range of highly functionalized systems [1–4]. The detailed computational experience was taken from using the most advanced wave function methods such as the most popular one, density functional theory (DFT). For highly functionalized systems such as pyridopyrimidines [1, 2, 4–6] triazole, and [1–4] tetrazole derivatives, these types of compounds are interesting for different biological applications such as anti-microbial and cytotoxic activities [7]. Several studies are concerned with the causes and solving different types of cancer problems and how to limit their occurrence. Low molecular weight ligands, such as heterocyclic Schiff bases, can interact with protein receptors demonstrating an effective binding affinity. The docking score function in ligand-protein interaction evaluates several non-covalent interactions [8–10] that may be considered a weak type compared with covalent bonds. However, the former type between the ligands and amino acids of protein gives some interesting information about regulation and enzyme transformation in bioinformatics and drug design [11–13]. Other more, most heterocyclic compound classes have been studied to explore the role of strong chelating ligands with a variety of transition metals based on electron-rich sites [14, 15].

To implicate the role of heterocyclic ligands against severe acute respiratory syndrome coronavirus 2 (SARS-CoV-2), a set of similar functional compounds was experimentally studied against this severe virus to enrich and become the main support in investigating a spectral range of similar compounds [16, 17].

The current study aimed to investigate the geometrical structural, and electronic molecular properties of five previously synthesized in our laboratory and characterized heterocyclic ligand compounds [18]. DFT computational study can interpret these structures according to the nature of each branched residue in the parent part. Electronic circular dichroism can find keywords about the definition of chiroptical properties and degree of absolute configuration in solutions, besides the importance of UV-Vis spectroscopy. Additionally, researchers direct their interest to molecular docking studies to illustrate the binding effect and related it to the experimental biological activities exploring the more potent ligand effect in treating several intractable diseases. From the previous experimental work, biological studies were applied for anticancer activity and exported reliable results. In this paper, we noticed the important value of study with the viral enzymes predicting a future positive inhibition effect. Molecular simulation against SARS main protease viral enzyme (Mpro) may be helpful as a point of support in a future experiment comparison. A spectral series of organic compounds were previously predicted as a good inhibitor for viral activity especially SARS-Cov-2 [19, 20]. Several heterocyclic systems play important role as anti-bacterial, anti- viral, anti-fungal, anti-tumor, and anti-covid-19 agents as well [21–23]. Coronavirus belongs to the family of viruses having a single stranded-RNA genome and capable of causing mild to severe symptoms of respiratory distress Molecular dynamic simulation analysis also become a strong support in evaluating the free energies of binding besides helpful indices to direct our mind the effective protein binding sites.

The studied heterocyclic compounds namely arranged as 6-(1-Methyl-1H-pyrrol-2-yl)-4-thiophen-2-yl-3,4-dihydro-1H-pyrimidine-2-thione (L1), 6-(1-methyl-1H-pyrrol-2-yl)-3-phenyl-8-(thiophen-2-yl-2,3-dihydro-8H-isoxazolo[5ˋ,4ˋ:4,5]thiazolo[3,2-a]pyrimidine (L2), 7-(1-Methyl-1H-pyrrol-2-yl)-3-phenyl-5-thiophen-2-yl-5H-[1,2,4]thiadiazolo[4,5-a]- pyrimidine (L3), 5-Methyl-2-[4-(1-methyl-1H-pyrrol-2-yl)-6-thiophen-2-yl-1,6-dihydro-pyrimidin-2-yl]-2,4-dihydro-pyrazol-3-one (L4), 7-(1-Methyl-1H-pyrrol-2-yl)-5-thiophen-2-yl-1,5-dihydro-[1,2,4]triazolo[4,3-a]- pyrimidine (L5).

## Method of calculations

### Computational study

Computational studies using DFT were applied to a certain class of non-substituted synthesized heterocyclic compounds (ligands) L1, L2, L3, L4 and L5, and significant optimized geometrical and electronic calculations were obtained based on density functional theory (DFT) using (B3LYP) method [24, 25]**,** with the basis set 6-31G (d,p) using Gaussian 09 software [26] and the calculations were initiated in presence of ethanol as a solvent. Geometrical parameters (bond lengths and bond angles) were calculated for the optimized structures and some important quantum molecular descriptors were calculated describing the electronically excited levels of the system. The charge on each atom was calculated using natural bond orbital (NBO) analysis. In order to evaluate the important electrophilic and nucleophilic sites for the optimized structures, MEP analysis was performed. Also, Avogadro^'s^ software [27] was used to sketch the ligands structures under theoretical study in addition to using Gauss view 06 [28] to visualize the optimized structure as well as the frontier molecular orbitals (FMOs). IR spectral analysis of the fully optimized heterocyclic compounds were calculated at the same DFT level considering the scale factor treated for B3LYP functional as the vibrational data generated without harmonic corrections. 1H, 13C NMR analyses were performed with the method of Gauge Including Atomic Orbitals (GIAO/DFT) [29].

Time dependent-DFT (TD-DFT) is a widely used method describing UV/vis spectra corresponding to the electronic transition states present in different compounds [30].

TD-DFT was performed concerning with the conductor-like polarizable continuum model (CPCM) in ethanol as a solvent. Additionally, ^1^H, ^13^C NMR spectral analyses were applied prior optimization using the gauge-including-atomic-orbital (GIAO) method.

### Molecular docking

Choosing the target protein for antiviral activity based on the ligand affinity probabilities and competitions between several docked poses using the suitable docking parameters. 6WTT-macromolecule crystal structure (resolution 2.15 Ǻ) was studied by M. Chunlong et al. [31] investigating a series of promising inhibitors to the protein activity. The optimized molecules (L1–L5) have been docked into the active site of Mpro enzyme as a receptor (6WTT, chain A and B) to study the strength of interaction occurring and to find a theoretical correlation with their COVID-19 antiviral activity. The antiviral activity of the reference promising inhibitor GC-376 (also, mentioned as K36/PRD_002495 in the dictionary of biologically interesting molecule reference BIRD https://www.wwpdb.org/data/bird) was used as a standard for virtual docking analysis. Molecular docking study was performed by the aiding of iGemdock 2.1 software [32]**.** The selected target protein was firstly prepared by removing water molecules, additional ions and ligands not planned in the study protocol. Polar hydrogens were added to protein and also Gasteir charges were assigned. Docking accuracy sittings include genetic algorithms (GA parameters) include size of 200 populations and selecting 70 generations with number of solutions equal 2. The ligand intra-energy is the major contributor of docking score function that select the binding mode and site of binding to the protein relative to the promising GC-376 antiviral activity. GemDock scoring function was applied based on multi-defined potential energy function deriving the binding site from the bounded reference ligand and then make a specific competion with the trained or studied compounds resulting a more docking accuracy. BIOVIA Discovery Studio Visualizer v21.1.0 software (http://www.accelrys.com) and Chimera 1.13.1 [33] which was used for visualization and specifying the types of nonbonding interactions originated from the docking tool. The crystal structure of main protease viral enzyme was obtained from (PDB) the Protein Data Bank (www.rcsb.org) considered as a target protein in this study. Molecular dynamic simulation was applied on the current heterocyclic ligands based on the solvated model TIP3PBOX hat refer to transferrable intermolecular potential of three-point grid box. A step of charge neutralization followed by energy minimization for the bio-complexed structure was applied. The five ligands were solvated based on TIP3PBOX model that refer to transferrable intermolecular potential of three-point grid box. A step of charge neutralization followed by energy minimization for the bio-complexed structure was applied. For each system, a total 100 ns simulation was achieved.

## DFT Results and discussion

### Geometrical structures

**Fig. 1:**
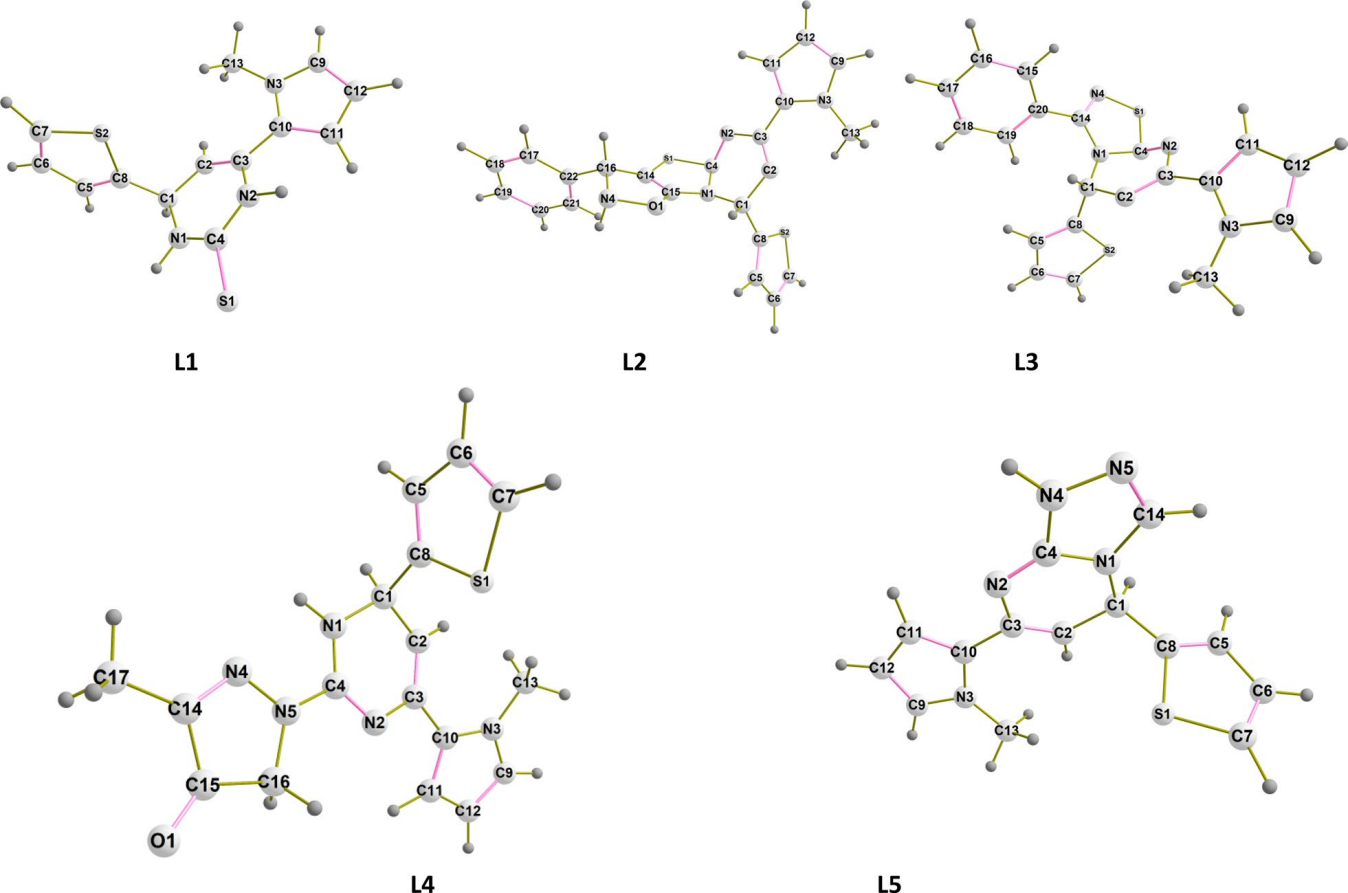
Optimized structures of the selected heterocyclic ligands with DFT/B3LYP/6-31G (d,p) method (pink colored bonds refer to double bonds in each compound)

The molecular optimized structures of the selected our previously synthesized ligands (L1–L5) were assumed with labelling of atoms using B3LYP /6-31G (d,p) in ethanol as a solvent, as shown in Fig. 1. The optimum bond lengths and bond angles of the selected ligands are tabulated at Additional file 1: Table S1. Our focus was directed to the substituent attached to the common center (C4) that can be changed from heterocyclic ligand to other. It is clear from that in the case of ligand L1, the bond length of C4-S1 is 1.703 Å, which is a value close to the value of the single bond length, while in case of the ligand L2, the C4-S1 bond length is 1.875 Å also in case of the ligand L3, the C4-S1 bond length is 1.839 Å assured that they are pure single bonds. C4-N5 and C4-N4 bonds have the values 1.387 Å and 1.364 Å for both L4 and L5, respectively. The slight difference may be attributed to the restricted structure of ligand L5 where C4-N4 is included in a cyclic five-membered ring while in case of ligand L4, C4-N5 has the ability of free rotation, that can affect on the bond length and structure conformation.

### Quantum and molecular reactivity study based on TD-SCF level

Highest and lowest-unoccupied molecular orbitals (HOMO and LUMO) energies were calculated for the optimized tested heterocyclic compounds (L1-L5) using the method of gradient energy analysis in the excited state applying time-dependent self-consistent field (TD-SCF) electronically excited level in ethanol as a solvent. Some of the important reactivity indices were derived from energy values of HOMO and LUMO such as chemical hardness (η), ionization potential (I), electron affinity (A), chemical potential (μ) and global chemical softness (σ), total molecular energy (E a.u.) and dipole moment (D) [34] as shown in Additional file 1: Table S2. These calculated parameters, such as dipole moment (D) can give information about the ability of charge delocalization on the molecule in solutions; for example, ligand L1 has a higher dipole moment (6.85 debye) so that it has a higher ability to form polar structure, while the less charged contributing species is represented by ligand L5 with low dipole moment (3.83 debye). In general, these calculated parameters play an important role in illustrating the effect of ligands interaction when undergoing molecular docking with a binding pocket like COVID-19 Mpro enzyme [35].

### FMOs and NBO

**Fig. 2:**
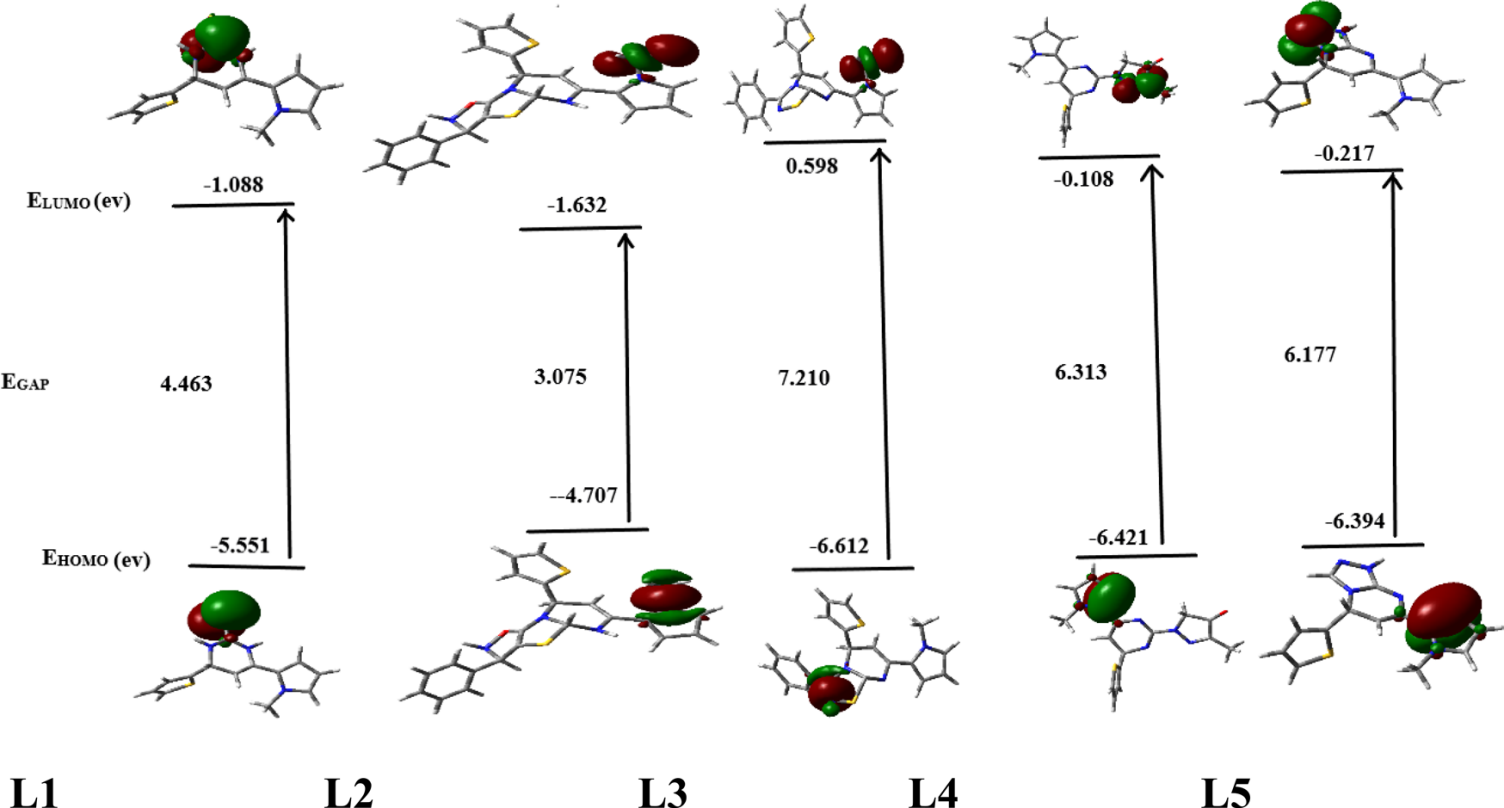
FMOs distribution energy structures of the optimized heterocyclic ligand compounds

**Fig. 3:**
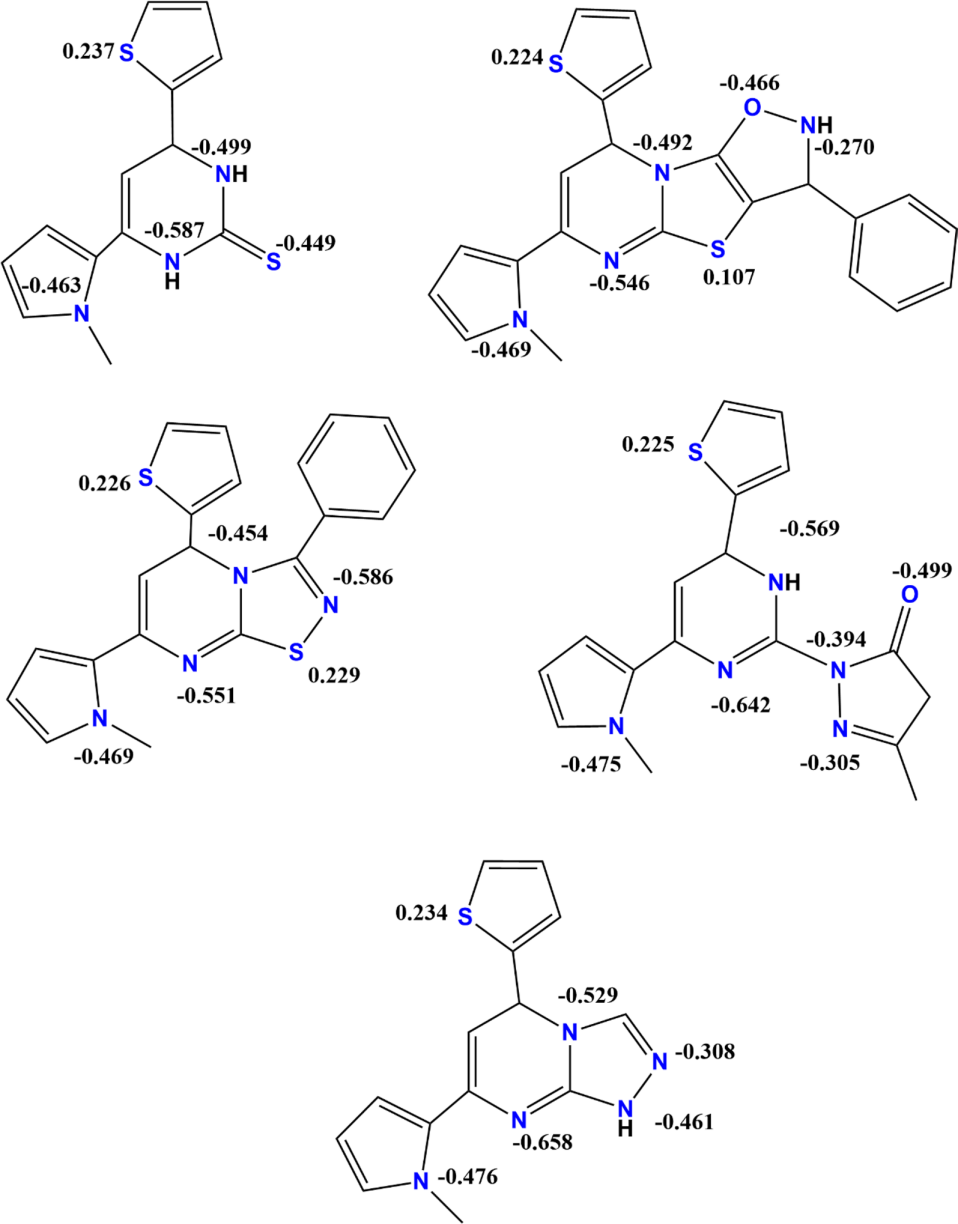
2D- structure of the studied compounds with charges on each heteroatom using DFT/ B3LYP method

The study of the frontier molecular orbitals (FMOs) properties of compounds is very important to predict the reactivity and stability of different molecules. As Fig. 2 illustrates the energy distribution of FMOs for the optimized compounds in ethanol. The energy gap (E_GAP_) is a suitable value discuss how stability of these compounds reach in solutions. L1 and L2 are predicted to be formed with E_GAP_ values of 4.463 eV and 3.075 eV, respectively, with ease of stable conditions. While L3, L4 and L5 are predicted to be formed with EGAP ≥ 6.177 eV and the time parameter may affect on their formation and stability. Also, electron delocalization between FMOs in the studied ligands mainly present on nitrogen atoms, pentacyclic π-bond of the compounds and thione group of ligand L1. In conclusion, there is a gradient in the of energy gap values leading to difference in the compound chemical reactivity. Figure 3 shows the 2D- scheme of the studied compounds with charges on each heteroatom resulting from the analysis of NBO calculation in ethanol. Charge calculations are very useful in predicting the electron-rich and electron-poor sites in the molecule that lead to the determination of the electrophilic and nucleophilic centers of the molecule. In ligand L1, the higher negative charge on N1, N2 and S1 leads to act as nucleophilic centers compared with N3 and S2. Also, in ligand L2 the electronic charge distribution appears on the 5-membered N3-cyclic ring. In ligand L3, the electron-rich center present on N1 and N4 of the 5-memebered ring, but not on the S atom which is less electronegative than nitrogen atom. The electrophilic centers mainly represent nitrogen atoms in L4 and L5.

### Computational comparison for spectroscopic properties

#### IR spectral analysis

Vibrational IR spectra of the studied ligands were calculated to ensure their molecular structures.

**Fig. 4:**
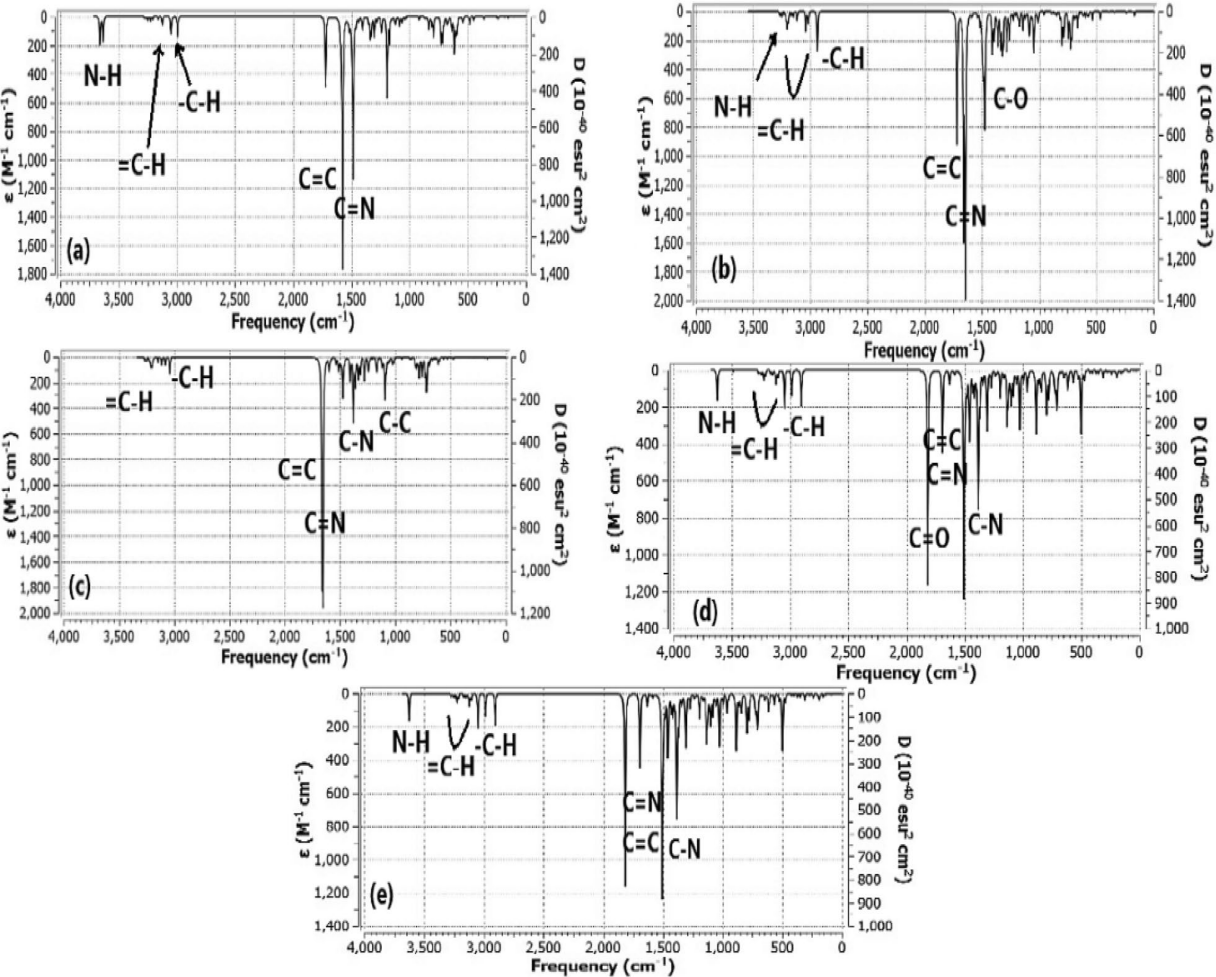
Calculated IR spectra of heterocyclic compounds at B3LYP/6-31G (d,p) level where. **a** compound L1, **b** compound L2, **c** compound L3, **d** compound L4, **e** compound L5

Quantum mechanical methods are based mainly on harmonic potentials, there are a step of correction should be done before comparing the computational with experimental results. It was approximated that the data investigated should be multiplied in a scale factor 0.962 to be calibrated with the experimental results (https://cccbdb.nist.gov/vibscalejust.asp). Table 1 shows the data corrected with the scale factor specified for B3LYP functional and also present the corresponding experimental data for each studied ligand. Some characteristic peaks for the five ligands appeared in Fig. 4. Because ab initio methods deal with harmonic vibrations without anharmonic corrections, the scale factor should be considered according to the basis set applied. Frequency values were multiplied in 0.962 and the data were collected at Table 4 with experimental results. Spectroscopic vibration of compound L1 shows two bands at 3519 cm^−1^ and 3497 cm^−1^ which corresponding to NH groups, the experimental results show the two peaks at 3368 cm^−1^, 3243 cm^−1^, this difference may be attributed to the presence of sulfur atom near NH groups which decrease the vibrational frequency. Small bands appear at 3140 cm^−1^ that corresponding to = CH and another band appear at 3041 cm^−1^ that corresponding to –C–H alkyl stretching group. C = C and C = N sharp bands appear at 1561 cm^−1^ and 1437 cm^−1^, respectively. Experimental IR band of C = C appear in range (1612–1602) cm^−1^ and this result is closer from the theoretical one. In case of compound L2, a band appears at 3357 cm^−1^ corresponding to NH group, small bands appear at 3161 cm^−1^ that corresponding to = CH and another band appear at 2997 cm^−1^ that corresponding to –C–H group. C = C and C = N sharp bands appear at 1597 cm^−1^ and 1581 cm^−1^, respectively [18]**.** A sharp band present at 1434 cm^−1^ corresponding to C–O group. For compound L3, small bands appear at 3161 cm^−1^ that corresponding to = CH and another band appear at 3030 cm^−1^ that corresponding to –C–H group. C = N and C = C sharp bands appear at 1599 cm^−1^ and 1592 cm^−1^, respectively. Small bands present at 1432 cm^−1^–1329 cm^−1^ corresponding to C–N group. C–C group appeared at 1124 cm^−1^. In case of compound L4, a small band appears at 3487 cm^−1^ that corresponding to –NH and another band appear at 3150 cm^−1^ that corresponding to = CH SP^2^ stretching, the band at 3047 cm^−1^ that corresponding to –C–H SP^3^ alkyl stretching. The band appears at 1647 cm^−1^ is corresponding to C = O group. C = C and C = N sharp bands appear at 1628 cm^−1^ and 1569 cm^−1^, respectively. For compound L5, a small band appears at 3564 cm^−1^ that corresponding to –NH, small bands appear at 3165 cm^−1^ that corresponding to = CH and another band appear at 3033 cm^−1^ that corresponding to -C-H group. C = N and C = C sharp bands appear at 1644 cm^−1^ and 1587 cm^−1^, respectively. Most of computational data evaluated agree with the experimental results [18]**.**

**Table 1:** Calculated frequencies from B3LYP/6–31(d,P), corrected values treated with scale factor (0.962), and experimental IR frequencies

Functional group	Frequency B3LYP/6–31(d,p) (cm^−1^)	Frequency x scale factor (cm^−1^)	Experimental Frequency (cm^−1^)
L1			
N–H	3659 and 3634	3519 and 3497	3368 and 3243
= C–H	3265	3140	3050
–C–H	3162	3041	2978
C = C	1623	1561	1612–1602
C = N	1494	1437	1488
L2			
N–H	3490	3357	3225
= C–H	3286	3161	3060
–C–H	3116	2997	2989
C = C	1661	1597	1615–1589
C = N	1644	1581	1619
C–O	1491	1434	1491
L3			
= C–H	3286	3161	3058
–C–H	3150	3030	2967
C = N	1663	1599	1624 and 1631
C = C	1655	1592	1608–1599
C–N	1489–1382	1432–1329	1490
C–C	1169	1124	1100
L4			
N–H	3625	3487	3195
= C–H	3275	3150	3069
–C–H	3168	3047	2988
C = O	1816	1647	1691
C = N	1693	1628	1631, 1625
C = C	1631	1569	1609–1595
C–N	1490–1373	1433–1320	1496
L5			
N–H	3705	3564	3275
= C–H	3290	3165	3060–3030
–C–H	3153	3033	2923
C = N	1709	1644	1643, 1632
C = C	1650	1587	1611–1594
C–N	1492–1377	1324–1435	1493

#### ^1^H and ^13^C NMR spectral investigation

**Fig. 5:**
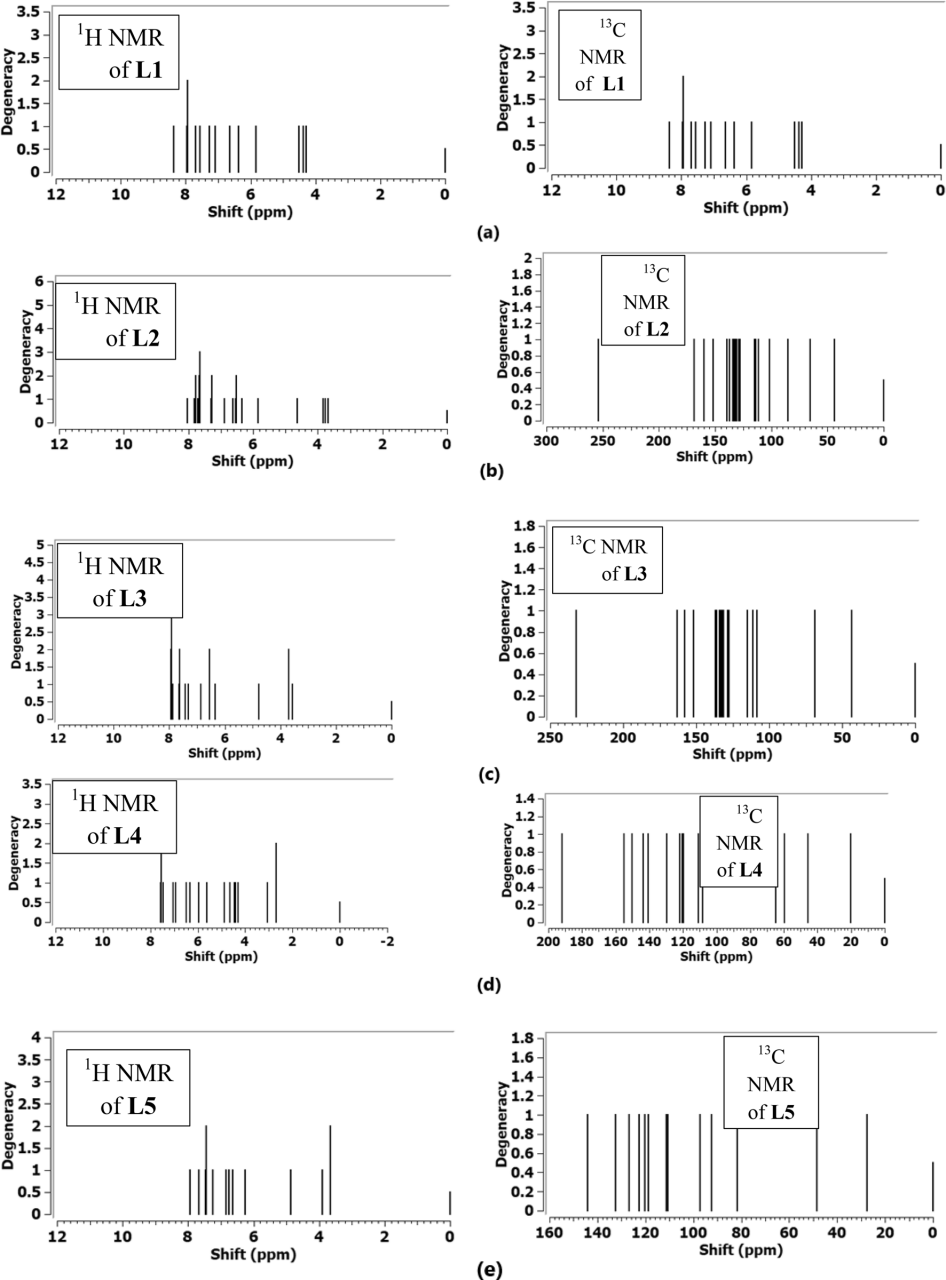
^1^H-NMR and ^13^C-NMR spectra of compounds L1, L2, L3, L4 and L5 using GIAO method on the optimized heterocyclic structures

Specific NMR spectral method of analysis, GIAO-SCF, was applied on the optimized B3LYP/ 6-31G (d,p) novel heterocyclic compounds in DMSO as a solvent. Figure 5 shows ^1^H and ^13^C NMR spectral analysis of the studied heterocyclic compounds. In case of ^1^H NMR of **L1** ligand, the position of two peaks at 6.64 ppm and 7.55 ppm while experimentally it appears at 8.89 ppm and 9.78 ppm, the difference may be attributed the solvent effect in formation non covalent bonds with NH. Also, for H4-pyrolle, its position appears at 7.27 ppm matching the reported experimental data 7.09 ppm [18]. Pyrimidine and thiophene hydrogens show a similarity with the experimental as present in the benchmark Additional file 1: Table S3. With ^13^C NMR spectral data, NCH_3_ appeared at 42.97 ppm while the reported experimental value presents at 39.57 ppm. C4 of pyrimidine appears at 62.67 ppm and experimentally is shown at 65.37 ppm [18]. In case of L2 ligand, H-5 of pyrimidine appears at 6.62 ppm and this is closer to the experimental data (6.68 ppm). Also, hydrogens of phenyl ring experimentally range from 7.43 to 8.11 ppm, while B3LYP estimated the peaks at 7.27–8.05 ppm and these values matches the reported ones. 3H of NCH_3_ appear as three peaks at 3.69 ppm, 3.75 ppm, 3.84 ppm 3.91 ppm and experimentally appear at 3.38 ppm. 3H of pyrrole appear in the experimental range 6.63 ppm–7.23 ppm and computationally appear at 6.35 ppm, 6.52 ppm, 6.89 ppm. For 13C NMR spectral data, carbon of NCH_3_ give peak at 43.79 ppm matches with the reported value (45.44 ppm). Carbon peak of C = N appears at 151.78 ppm and experimentally present at 161.10 ppm. ^1^H NMR and ^13^C NMR spectral analysis of the other L3, L4 and L5 compounds show different line positions according the environment surrounded the atoms and most of data are closer to the reported experimental results [18].

#### Benchmark ^1^H- and ^13^C- NMR analysis with different DFT-ab initio functionals

To estimate the more accurate functional used in structure description, a comparison was made for some available functionals such B3LYP, BPV86 and B3PW91 methods. Additional file 1: Table S3 presents in details, the data of ^1^H and ^13^C NMR obtained for the five heterocyclic compounds by using the three computational functionals. A helpful investigation to suggest using the suitable computational method is to estimate the position of the peak and determine its chemical shift δ (ppm), then a systematic comparison around the experimental results was applied. The functionals performed in this study are B3LYP, BPV86 and B3PW91 methods. The observed H-NMR data responsible for NH by the three computational methods is bad estimated as experimentally appeared around 9.98 ppm (i.e. L1) but theoretically appeared around 6.53 ppm, 6.96 ppm and 8.31 ppm for B3LYP, BPV86 and B3PW91 methods, respectively. The best experimentally matching H-NMR values of pyrrole ring devolved to B3LYP method. Hydrogen of isoxazole is bad estimated with BPV86 as it appears at 7.27 ppm while experimentally, appears at 5.83 ppm. Also, 13C NMR spectra were obtained and a detailed comparison can be interpreted from Additional file 1: Table S3. Most of the data observed from B3LYP match well with experimental results rather than other studied functionals.

#### UV–Vis electronic spectra using TD-DFT method

**Fig. 6:**
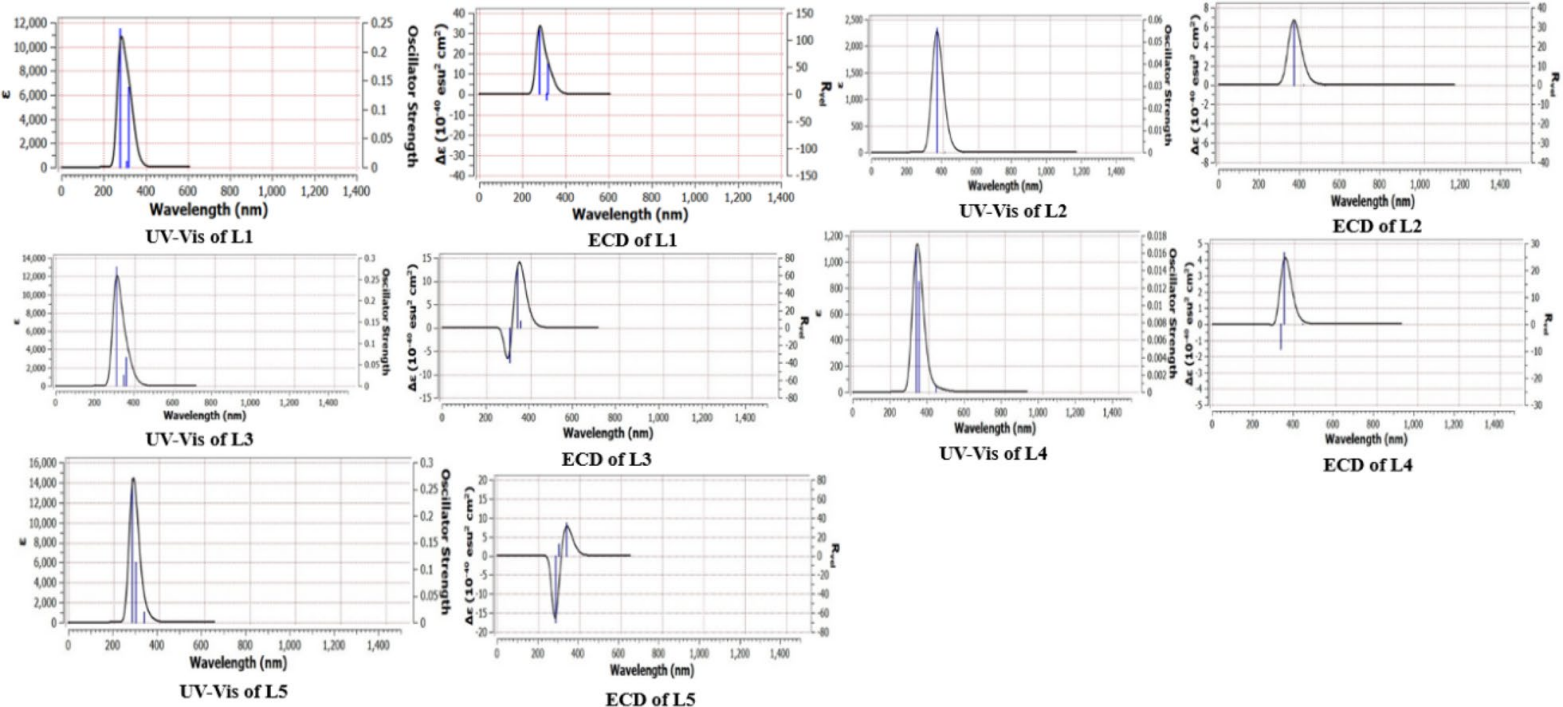
UV–Vis and ECD electronic absorption spectra of the studied heterocyclic compounds

The default settings of Gaussian software were performed for TD-DFT with CPCM solvation model calculations. The program settings were adjusted for N state = 6 to analyze of six states. To examine the electronic behavior of studied heterocyclic compounds, UV-visible peaks were observed for (L1-L5) at maximum wavelengths between 200 and 400 nm as shown in Fig. 6. In case of L1, the detailed LOG gaussian file give an imagine about the first transition which represents a singlet strong absorption band corresponding to HOMO−2→LUMO transition with orbital contribution 61.7 % at λmax = 279.47nm. For L2, the absorption band strongly appears at λmax = 366.50 nm corresponding to HOMO→LUMO transition with orbital contribution 67 % For L3, there are three singlet transitions appeared corresponding to HOMO →LUMO, HOMO→LUMO+1 and HOMO−1→LUMO based on n−π* and π−π* electronic transitions. For L4, one weak and two strong transition peaks appeared at λmax of 439.27 nm, 349.07 nm and 334.06 nm. These transitions involve HOMO→LUMO, HOMO−2→LUMO and HOMO−1→LUMO based in n−π* and π−π* electronic transitions.

In case of L5, there are three transition spectra with λmax of 336.14 nm, 299.64 nm and 283.21 nm that corresponding to HOMO→LUMO, HOMO →LUMO+1 and HOMO−1→LUMO based on n−π* and π−π* electronic transitions. Table 2 shows the detailed UV-Vis electronic transition spectra with energies and oscillator strength data. Also, Figure 5 describes several electronic circular dichroisms (ECD) spectral lines of each studied heterocyclic ligand that give some information about isomerism and structure transformation. ECD spectra of L1 shows two strong (+) camphor and one weak (−) estimated isomeric camphor. L2 appeared as one (+) camphor, while L3 shows (+) and (−) camphor as in case of L5. L4 shows strong singlet (+) line and a weak singlet (−) line. The difference in peak position and strength indicates the different molecular configuration structure.

**Table 2:** Excitation energies, maximum wavelengths, oscillator strengths and % orbital contribution for the computationally studied compounds

Compound	Spectral line number	Excitation energy (eV)	λ_max_ (nm)	F	Type of transition	% Orbital contribution
L1	1	3.883	319.26	0.138	HOMO → LUMO	66.70
	2	3.989	310.81	0.010	HOMO−1 → LUMO	58.28
	3	4.436	279.47	0.238	HOMO−2 → LUMO	61.7
L2	1	3.318	518.16	0	HOMO → LUMO	61.04
					HOMO−1 → LUMO	16.70
	2	3.002	412.98	0	HOMO → LUMO	18.00
					HOMO−1 → LUMO	58.36
	3	3.383	366.50	0.056	HOMO → LUMO	67.00
					HOMO → LUMO + 2	13.71
L3	1	3.497	354.53	0.067	HOMO → LUMO	69.43
	2	3.627	341.8	0.025	HOMO−1 → LUMO + 1	15.11
					HOMO → LUMO + 1	67.14
	3	4.053	305.91	0.278	HOMO−1 → LUMO	60.82
					HOMO−1 → LUMO + 1	31.70
L4	1	2.822	439.27	0.0006	HOMO → LUMO	70.24
	2	3.552	349.07	0.013	HOMO−1 → LUMO	18.00
					HOMO−2 → LUMO	49.02
	3	3.711	334.06	0.016	HOMO−1 → LUMO	67.03
					HOMO−2 → LUMO	14.47
L5	1	3.688	336.14	0.019	HOMO → LUMO	67.23
					HOMO → LUMO + 1	19.04
	2	4.137	299.64	0.112	HOMO → LUMO	16.02
					HOMO → LUMO + 1	65.00
	3	4.378	283.21	0.269	HOMO−1 → LUMO	67.37
					HOMO → LUMO + 1	13.26

### Molecular electrostatic surface potential (MEP)

**Fig. 7:**
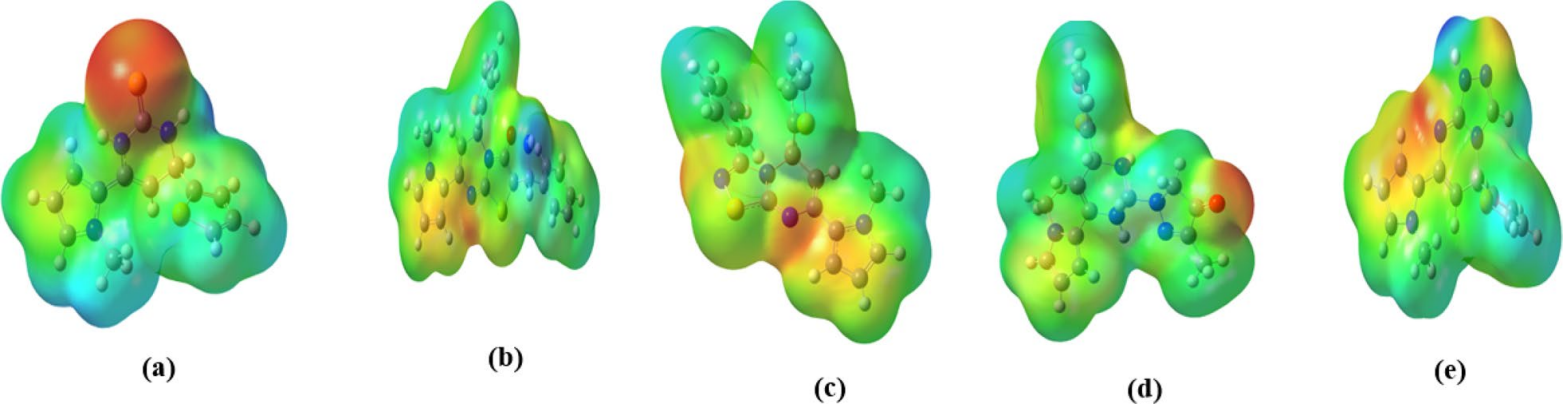
MESP map of the optimized heterocyclic structures, **a** L1, **b** L2, **c** L3, **d** L4, **e** L5, where color mapped from red (highly negative charge value) to blue (highly positive charge value)

MEP describes electrostatic potential mapped to the surface of a constant electron density and mainly used to predict the relative molecular sites reactivity towards electrophilic reactions in the studies responsible for biological recognition and H-bond interactions [36]. Figure 7 shows the mapped MEP scheme for the studied optimized heterocyclic ligands (L1–L5) where the positive electrostatic surface potential (ESP) corresponds to the lower electron density regions that colored in blue while the negative ESP corresponds to the higher electron density regions that colored in red. In ligand L1, the red (negative) region is localized on the sulfur atom (S9) and a slight negative charge on N3 and N5 while the blue (positive) region appears mainly on the methyl substituent of azole group. In case of ligand L2, the electron density centers comprise the azole group and N3 while the positive site is represented in the phenyl group of the molecule. The N3 and azole group in ligands L3 and L5, as well as O37 of ligand L4 are considered as the highly electronic centers, but there are many positive sites in the ligands L3, L4 and L5.

The average local ionization energy (ALIE) exports an important role in describing the electronic surface of molecules. ALIE is the energy necessary to remove the electron from a point r in the system. Electrophilic behavior can be shown from the color code evaluated from the ALIE map in Additional file 1: Fig. S1. The blue color with cyan points describes the electron rich sites for the studied systems. Fukui function analysis was performed to investigate the nucleophilic and electrophilic active sites. Fukui function with parameters *fk*^–^ and *fk*^+^ can represent the electron density of HOMO (electrophilic sites) and LUMO (nucleophilic sites), respectively. Additional file 1: Tables (S4–S8) shows the values originated from this analysis. The electrophilic and nucleophilic spots are marked with bold for the five heterocyclic systems.

### Virtual molecular docking results

**Fig. 8:**
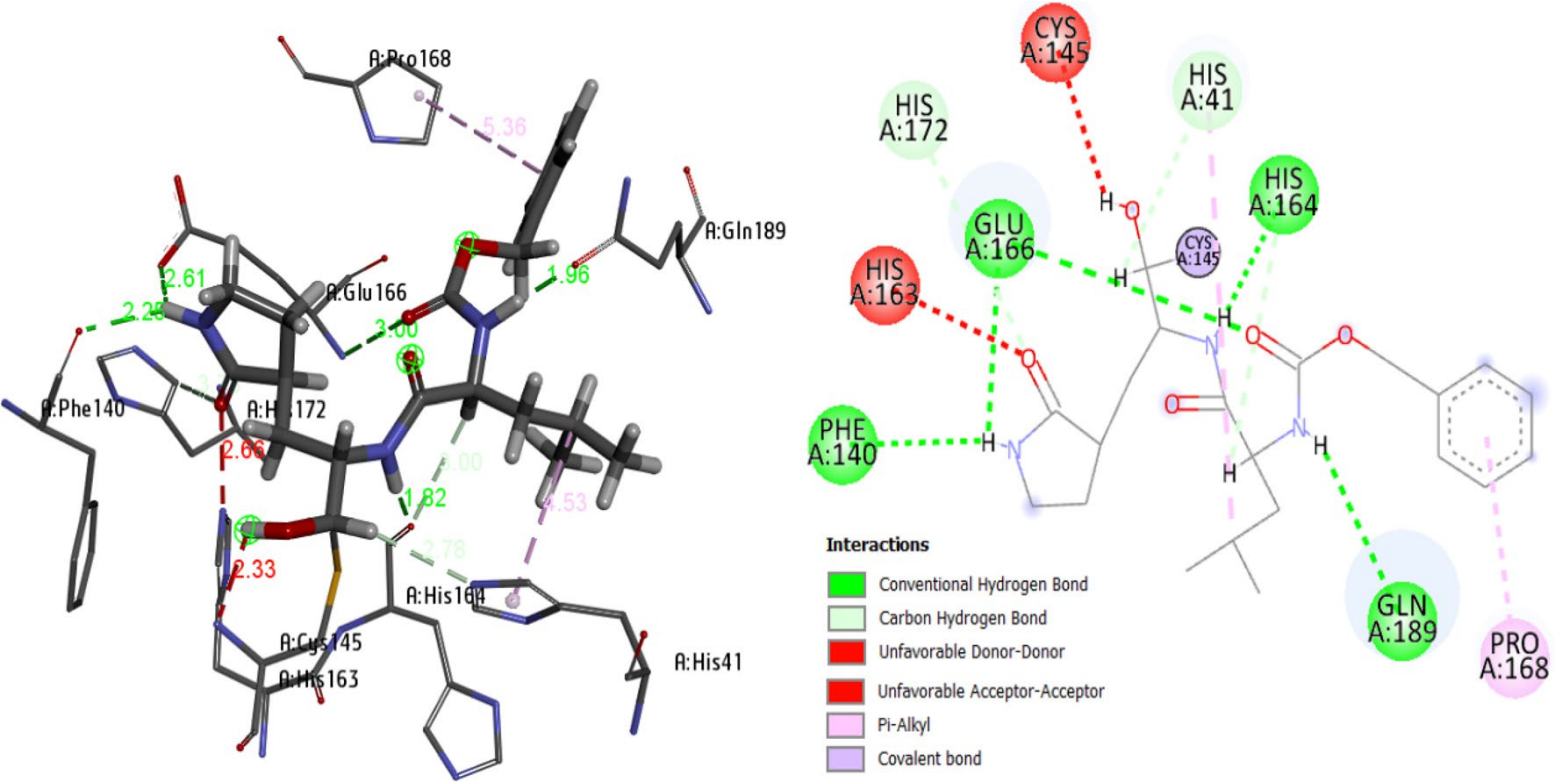
K36 Reference ligand inhibitor in Mpro enzyme with PDB 6WTT

The strength of interactive poses should be mainly estimated through the type of interaction to form an effective bio-complexed system [37]. In order to estimate the most effective inhibitory ligand structure on viral activity, the total binding energy, H-bond energy and VDW energy of the studied ligands, in the optimized state, were calculated as shown in **Table ****3**. Also, A series of the interacted studied ligands with different types of amino acids through certain distances compared with the reference inhibitor k36 ligand was shown in Table 4, where the interacted reference structure of K36 was presented in Fig. 8. We conclude from the docking results that the investigated ligands have closely similar binding energy except a very slight variation. L2, L3, L5 a high binding energy (− 98.210 kcal/mol, − 98.678 kcal/mol, − 98.993 kcal/mol) and as a result have a suitable predicting binding affinity with Mpro enzyme receptor. As shown in Fig. 7, the reference ligand k36 interacts with amino acids of chain A through various types of interactions. For example, PHE.A 140, HIS.A 164, GLU.A 166 and GLN.A 189 are interacting with k36 via conventional H-bonding, while HIS.A 41, HIS.A 164 and HIS.A 172 via carbon hydrogen bond (i.e. non-conventional bond) and PRO.A 168 via π-alkyl interaction. Also as shown in Table 4, ligand L1 (i and i1) interacts with GLY.A 143 and HIS.A 164 receptors with H-bonding, while interacts with HIS.A 41 via π-sulfur interaction, along with the interaction of Van der Waals with a number of receptor amino acids. Ligand L2 (ii and ii1) interacts with GLU.A 166 via H-bonding while interacts with CYS.A 145 and MET.A 165 via π-sulfur interaction as well as alkyl and π-alkyl, amide- π stacked interactions with PRO.A 168, THR.A 190 and ALA.A 191. Docking of ligand L3 (iii and iii1) with Mpro enzyme gives different types of interactions such H-bonding, π-sulfur, alkyl and π-alkyl with attractive 11 amino acids. The observed interactions of ligand L4 (iv and iv1) are H-bonding with GLY.A 143 and two bonds with GLN.A 189, alkyl, π-alkyl interaction with HIS.A 41, CYS.A 145, MET.A 165, PRO.A 168 and VDW interaction with a number of amino acids. LEU.A 141, GLY.A 143, CYS.A 145 and GLU.A 166 interacts with ligand L5 (v and v1) via H-bonding, beside a number of binding sites interact through VDW. As H-bonding is the strongest non-covalent bond, this type can predict the effective inhibitors studied besides other attractive forces. The behavior of most studied compounds was predicted to act closely inhibited as k36 as the poses become compatible around the active site.

**Table 3:** Molecular docking distribution of the score fitting the optimized heterocyclic with Mpro enzyme (PDB: 6WTT)

Compound	Total Binding Energy (kcal/mol)	H-Bond Energy (kcal/mol)	VDW Energy (kcal/mol)	Molecule-Solvent Accessibility (kcal/mol)
L1	− 73.623	− 4.109	− 69.513	3310.40
L2	− 98.210	− 92.210	− 6.00	2747.13
L3	− 98.678	− 85.678	− 3.212	3066.04
L4	− 91.403	− 82.590	− 8.813	2760.89
L5	− 98.993	− 75.108	− 23.885	2722.45

`

**Table 4:** **i**–**v**: diagrammatic 3D- and 2D-ligand-Mpro interaction in presence of k36 as a reference in the 3D-structure (K36 inhibitor is represented with purple color), **i1**–**v1**: non-covalent interactions of ligand with amino acids of Mpro

No	Diagrammatic 3D- and 2D-ligand-Mpro interaction in presence of k36 with non-covalent bond distance values	Non-covalent interactions of ligand with amino acids of Mpro
L1		
	(i)	(i1)
L2		
	(ii)	(ii1)
L3		
	(iii)	(iii1)
L4		
	(iv)	(iv1)
L5		
	(v)	(v1)

The docking study through Table 5 illustrates the different types of surface interactions between amino acids of receptor and the docked heterocyclic ligands, L1-L5, where the green regions on the surface of protein residues have the ability to accept hydrogen atom to form H-bonding means that these sites are electron rich, the pink-colored protein surface are electron poor, so the hydrogen atoms can be involved in H-bond with other electronic rich molecular species. In the case of hydrophobic effect, the surface of protein residue is stained blue which includes a number of hydrophobic interactions between the ligand and the residue represented in VDW, π-sulfur and π-alkyl bonds, whereas the regions scaled with brown are less hydrophobic and the probability of H-bond formation increase in these regions.

**Table 5:** H-bond and hydrophobicity of the target Mpro enzyme surface interacted with (**a**) ligand L1 (**b**) ligand L2 (**c**) ligand L3 (**d**) ligand L4 (e) ligand L5 (Ligand are represented by thick sticks and amino acids with thin sticks)

	
(a)	
	
(b)	
	
(c)	
	
(d)	
	
(e)	

### Molecular dynamic simulation (MDS) analysis

**Fig. 9:**
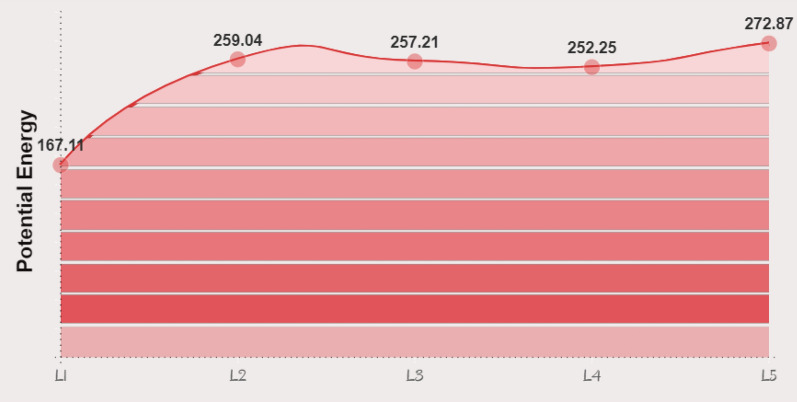
potential energy values of the studied heterocyclic compounds from molecular dynamic simulation

**Fig. 10:**
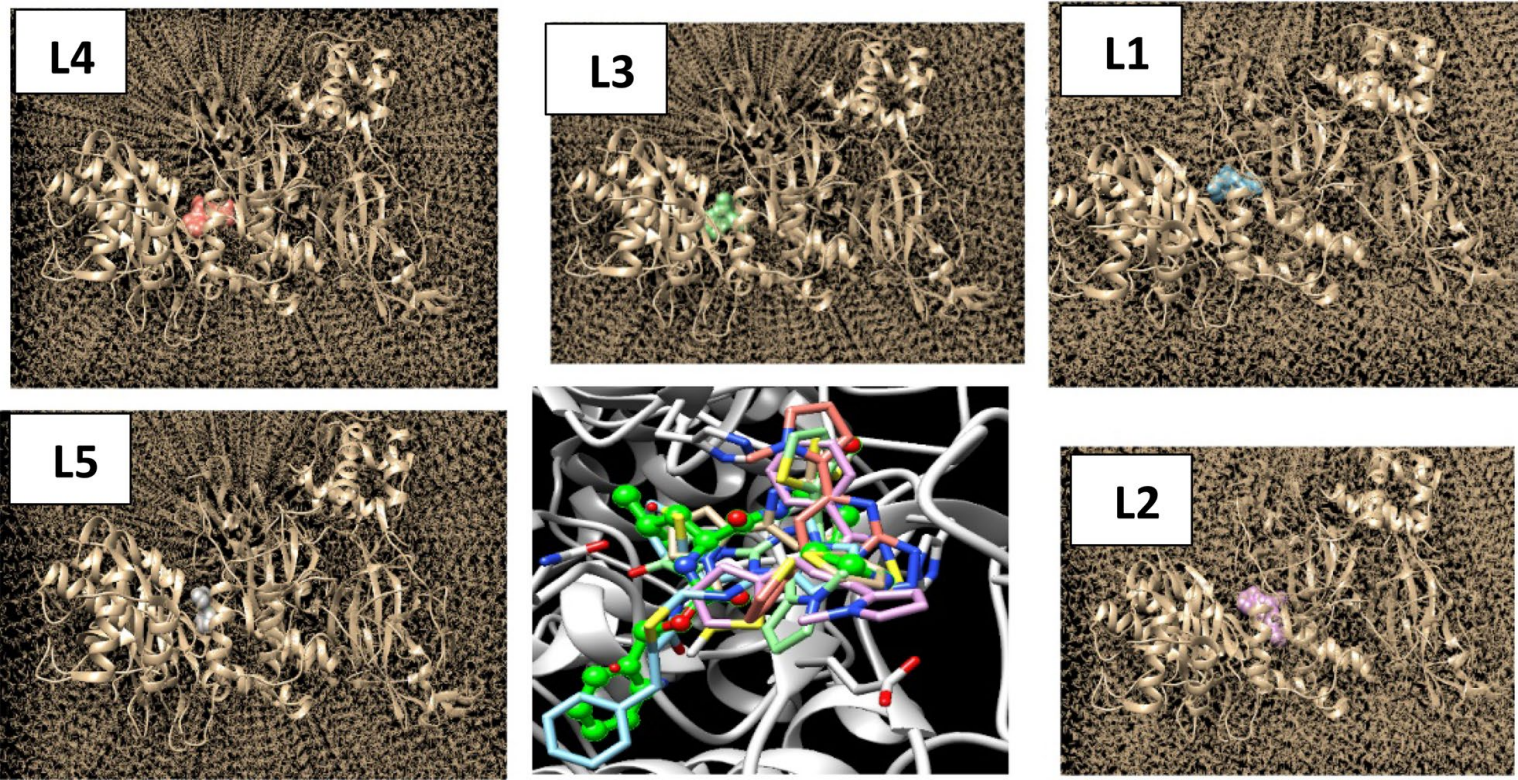
Solvation model of the target protein with each studied compound in the effective binding sites relative to K36 ligand (K36: green ball and stick structure)

Molecular docking accuracy is supported with MDS especially for compounds computationally studied without experimental evaluation. The process of dynamic simulation is related to the energy of poses in specific site with a fixed time. The studied systems were solvated based on TIP3PBOX model that refer to transferrable intermolecular potential of three-point grid box. A step of charge neutralization followed by energy minimization for the bio-complexed structure was applied. Binding affinity of the ligand in the more favorable protein site is measured according to the period which taken by the ligand to be fixed with interacted amino acids. Figure 9 shows the values of potential energy of each ligand in the final simulation step. The high potential energy values measure a good fixed ligand–protein period that allows a collection of interaction types with higher binding affinity. L5 shows a higher simulation result depending on the potential energy (272.87 kcal/mol). Root mean square deviation (RMSD) values predict the best pose of several ligand conformations obtained with respect to the position of atoms in the binding site. As predicted, most effective conformational poses were generated with RMSD values, 0.529, 0.326, 0.350, 0.437 and 0.154 for L1, L2, L3, L4 and L5, respectively. These values estimate the inhibitory effect of L5 compared with other studied ligands. In literature, RMSD values of small molecules should not be higher than 1 to achieve an inhibitory effect. So, these compounds still under the border range for antiviral consideration. Figure 10 represent the solvated target protein interacted with the ligands docked in the effective binding sites related to the reference inhibitor K36 (green ball and stick structure).

## Conclusion

Recently reported class of heterocyclic compounds were significantly characterized in details according to their geometrical and electronic framework. The spectral behavior of the studied pyrimidine‑2‑thiones based compounds matches the previously published experimental results and that ensure the best description of these compounds. Quantum chemical parameters differentiated the reactivity and stability of these heterocyclic systems through their molecular orbital energies occurred. Also, MEP analysis described the most electrophilic and nucleophilic sites present that initiate different reaction mechanisms in the future. Benchmark analysis was performed on NMR spectra with different computational functionals and investigated that method B3LYP is best describe the structure properties. The virtual docking of some chosen synthesized compounds L1, L2, L3, L4 and L5 was performed with Mpro viral enzyme protein in comparison with a k36 reference ligand inhibitor. The study indicated that the chosen compounds had the ability to form different types of interactions such H-bond and hydrophobic (VDW, π-alkyl and π-sulfur) interactions with Mpro enzyme receptor. Molecular dynamic simulation performance supported the molecular docking results in predicting the effective binding affinity of heterocyclic compounds.

## Supplementary Information

**Additional file 1: Table S1.** The optimum Geometrical parameters (bond lengths and bond angles) of the studied heterocyclic compounds. **Table S2.** Molecular reactivity parameters using TD-DFT-B3LYP/ 6-31G (d,p) of the studied heterocyclic compounds in solution. **Table S3.** Values of ^1^H, ^13^C NMR chemical shifts (ppm) of the studied by using heterocyclic compounds by using different ab initio functionals, and experimental data. **Table S4.** Values of the mulliken charges, Fukui function of L1. **Table S5.** Values of the mulliken charges, Fukui function of L2. **Table S6.** Values of the mulliken charges, Fukui function of L3. **Table S7.** Values of the mulliken charges, Fukui function of L4. **Table S8.** Values of the mulliken charges, Fukui function of L5. **Fig. S1.** ALIE of the heterocyclic systems (a) L1, (b) L2, (c) L3, (d) L4, (e) L5.

## Data Availability

The datasets generated and/or analyzed during the current study are available in the supplementary file.
