# Supplementary material for: Spectroscopic details on the molecular structure of pyrimidine‑2‑thiones heterocyclic compounds: computational and antiviral activity against the main protease enzyme of SARS-CoV-2
Source: BMC Chem. 2022 Nov 2;16(1):82. doi: 10.1186/s13065-022-00881-3 (PMC9628048; doi:10.1186/s13065-022-00881-3)
Supplement: Supplementary file 1 — Additional file 1: Table S1. The optimum Geometrical parameters (bond lengths and bond angles) of the studied heterocyclic compounds. Table S2. Molecular reactivity parameters using TD-DFT-B3LYP/ 6-31G (d,p) of the studied heterocyclic compounds in solution. Table S3. Values of 1H, 13C NMR chemical shifts (ppm) of the studied by using heterocyclic compounds by using different ab initio functionals, and experimental data. Table S4. Values of the mulliken charges, Fukui function of L1. Table S5. Values of the mulliken charges, Fukui function of L2. Table S6. Values of the mulliken charges, Fukui function of L3. Table S7. Values of the mulliken charges, Fukui function of L4. Table S8. Values of the mulliken charges, Fukui function of L5. Fig. S1. ALIE of the heterocyclic systems (a) L1, (b) L2, (c) L3, (d) L4, (e) L5. [file 13065_2022_881_MOESM1_ESM.docx]

**Table S1:** The optimum Geometrical parameters (bond lengths and bond angles) of the studied

heterocyclic compounds

| **L1** | | **L2** | | **L3** | | **L4** | | **L5** | |
| --- | --- | --- | --- | --- | --- | --- | --- | --- | --- |
| **Bond Length (Å)** | | | | | | | | | |
| C1-N1 | 1.480 | C1-N1 | 1.480 | C1-N1 | 1.483 | C1-N1 | 1.471 | C1-N1 | 1.477 |
| C4-N1 | 1.344 | C4-N1 | 1.463 | C4-N1 | 1.455 | C4-N1 | 1.356 | C4-N1 | 1.383 |
| C3-N2 | 1.411 | C3-N2 | 1.411 | C3-N2 | 1.409 | C3-N2 | 1.408 | C3-N2 | 1.405 |
| C4-N2 | 1.358 | C4-N2 | 1.454 | C4-N2 | 1.458 | C4-N2 | 1.300 | C4-N2 | 1.300 |
| C4-S1 | 1.703 | C4-S1 | 1.875 | C4-S1 | 1.839 | C4-N5 | 1.387 | C4-N4 | 1.364 |
| C9-N3 | 1.371 | C14-S1 | 1.784 | C9-N3 | 1.373 | N4-N5 | 1.356 | N4-N5 | 1.378 |
| C10-N3 | 1.387 | O1-N4 | 1.488 | C10-N3 | 1.387 | C7-S1 | 1.736 | C7-S1 | 1.736 |
| C8-S2 | 1.751 | C8-S2 | 1.751 | C8-S2 | 1.753 | C8-S1 | 1.752 | C8-S1 | 1.750 |
| C7-S2 | 1.736 | C7-S2 | 1.737 | C7-S2 | 1.737 | C15-O1 | 1.219 | C13-N3 | 1.457 |
| **Bond Angle (^o^)** | | | | | | | | | |
| N1-C4-S1 | 122.480 | N1-C4-S1 | 105.628 | N1-C4-S1 | 102.056 | N1-C4-N5 | 115.945 | N1-C4-N2 | 128.442 |
| N2-C4-S1 | 121.206 | N2-C4-S1 | 111.559 | N2-C4-S1 | 111.964 | N2-C4-N5 | 117.470 | N1-C4-N4 | 103.282 |
| C13-N3-C9 | 123.507 | C13-N3-C9 | 123.236 | C13-N3-C9 | 123.248 | C13-N3-C9 | 122.652 | N2-C4-N4 | 128.275 |
| C13-N3-C10 | 127.739 | C13-N3-C10 | 127.909 | C13-N3-C10 | 127.903 | C13-N3-C10 | 128.339 | C10-N3-C13 | 128.398 |
| C7-S2-C8 | 91.567 | C7-S2-C8 | 91.687 | C7-S2-C8 | 91.776 | C7-S2-C8 | 91.661 | C9-N3-C13 | 122.580 |
|  |  |  |  | S1-N4-C14 | 109.042 | N1-C4-N5 | 115.945 | C7-S1-C8 | 91.492 |
|  |  |  |  | N1-C14-N4 | 118.943 | N2-C4-N5 | 117.470 | C1-C8-S1 | 121.825 |

**Table S2:** Molecular reactivity parameters using TD-DFT-B3LYP/ 6-31G (d,p) of the studied heterocyclic compounds in solution

| **Parameter** | **L1** | **L2** | **L3** | **L4** | **L5** |
| --- | --- | --- | --- | --- | --- |
| **D** | 9.800 | 3.650 | 7.480 | 6.220 | 5.650 |
| **E_HOMO_ (ev)** | -5.551 | -4.707 | -6.612 | -6.421 | -6.394 |
| **E_LUMO_** | -1.088 | -1.632 | 0.598 | -0.108 | -0.217 |
| **E_GAP_** | 4.436 | 3.075 | 7.210 | 6.313 | 6.177 |
| **I^a^** | 5.551 | 4.707 | 6.612 | 6.421 | 6.394 |
| **A^b^** | 1.088 | 1.632 | -0.598 | 0.108 | 0.217 |
| **η ^c^** | 2.232 | 1.537 | 3.605 | 3.156 | 3.088 |
| **μ ^d^** | -3.320 | -3.170 | -3.007 | -3.264 | -3.305 |
| **σ^e^** | 0.448 | 0.650 | 0.277 | 0.317 | 0.323 |

**^a^I = - E_HOMO ,_ ^b^A = - E_LUMO,_ ^c^η = (I-A)/2, ^d^μ = - (I+A)/2, ^e^σ = 1/ η**

**Table S3.** Values of ^1^H, ^13^C NMR chemical shifts (ppm) of the studied by using heterocyclic compounds by using different ab initio functionals, and experimental data.

| **Experimental**  **δ (ppm)** | | **B3PW91/6-31G(d,p)**  **δ (ppm)** | | **BPV86/6-31G(d,p)**  **δ (ppm)** | | **B3LYP/6-31G(d,p)**  **δ (ppm)** | | **Atom** |
| --- | --- | --- | --- | --- | --- | --- | --- | --- |
| **^1^H NMR ^13^C NMR** | | **^1^H NMR ^13^C NMR** | | **^1^H NMR ^13^C NMR** | | **^1^H NMR ^13^C NMR** | |  |
| **L1** | | | | | | | | |
| 39.57 | 3.42 (S) | 41.44 | 3.65, 3.78, 3.88 | 44.23 | 3.74, 3.82, 3.95 | 42.97 | 4.30, 4.37, 4.51 | **^1^H NMR**  3 Hs in NCH_3_  **^13^C NMR**  C in NCH_3_ |
| 65.37 | 4.76 (d) | 60.87 | 6.05 | 65.22 | 6.07 | 62.67 | 5.83 | **^1^H NMR**  H-4 (pyrimidene)  **^13^C NMR**  C-4 (pyrimidene) |
| 108.30 | 6.33 (d) | 102.50 | 6.97 | 102.58 | 6.96 | 104.18 | 6.37 | **^1^H NMR**  H-5 (pyrimidene)  **^13^C NMR**  C-5 of pyrimidine |
| 119.86 - 130.18 | 6.95 (d) | 109.62 – 135.25 | 6.55 | 109.84 – 133.46 | 6.51 | 111.72, 127.55, 128.50, 136.52 | 7.09 | **^1^H NMR**  H-3 (pyrrole)  **^13^C NMR**  aryl carbons |
| 141.18 | 7.09 (dd) | 129.71 | 6.72 | 128.51 | 6.66 | 131.26 | 7.27 | **^1^H NMR**  H-4 (pyrrole)  **^13^C NMR**  C-6 of pyrimidine |
| 176.13 | 7.31 (d) | 174.59 | 7.14 | 167.78 | 7.06 | 177.55 | 7.90 | **^1^H NMR**  H-5 (pyrrole)  **^13^C NMR**  C=S |
| --- | 7.51 (d) |  | 7.42 |  | 7.40 | --- | 7.94 | **^1^H NMR**  H-3 (thiophene) |
| --- | 7.81 (dd) |  | 7.44 |  | 7.40 | --- | 7.97 | **^1^H NMR**  H-4 (thiophene) |
| --- | 8.17 (d) |  | 7.82 |  | 7.75 | --- | 8.36 | **^1^H NMR**  H-5 (thiophene) |
| --- | 8.89 (S), 9.78 (br) |  | 6.05, 6.97 |  | 6.07, 6.96 | --- | 6.64, 7.55 | **^1^H NMR**  2NH |
| **L2** | | | | | | | | |
| 45.44 | 3.38 (S) | 40.20 | 3.74, 3.77, 3.84 | 44.36 | 3.74, 3.81, 4.00 | 43.79 | 3.69, 3.75, 3.84 | **^1^H NMR**  NCH_3_  **^13^C NMR**  NCH_3_ |
| 64.22 | 5.56 (d) | 64.96 | 5.09 | 62.26 | 5.17 | 65.22 | 4.54 | **^1^H NMR**  H-4 of pyrimidine  **^13^C NMR**  C-4 of pyrimidine |
| 74.93 | 5.83 (S) | 76.96 | 6.37 | 81.79 | 7.27 | 85.23 | 5.85 | **^1^H NMR**  H-3 of isoxazole  **^13^C NMR**  C-3 of isoxazole |
| 123.55 – 149.32 | 6.68 (d) | 120.92 – 160.76 | 7.22 | 129.17 – 156.33 | 5.69 | 111.28-159.79 | 6.62 | **^1^H NMR**  H-5 of pyrimidine  **^13^C NMR**  C-aryl |
| 161.10 | (6.63-7.23) (m) | 155.39 | 6.73, 6.77, 7.24 | 160.26 | 6.43, 6.65, 6.95 | 151.78 | 6.35, 6.52, 6.89 | **^1^H NMR**  3H of pyrrole **^13^C NMR**  C=N |
| --- | 7.43 – 8.11 |  | 6.73 – 7.87 |  | 7.43 – 8.26 | --- | 7.27 – 8.05 | **^1^H NMR**  Hs of thiophene and phenyl |
| --- | 9.98 (br) |  | 8.31 |  | 6.96 | --- | 6.53 | **^1^H NMR**  NH |
| **L3** | | | | | | | | |
| δ 53.76 (NCH3 | 3.58 (S) | 41.32 | 4.14, 4.26, 4.30 | 44.01 | 4.20, 4.30, 4.35 | 43.66 | 3.57, 3.69, 370 | **^1^H NMR**  NCH_3_  **^13^C NMR**  NCH_3_ |
| 65.13 | 5.64 (d) | 63.13 | 5.61 | 67.62 | 5.60 | 68.77 | 4.79 | **^1^H NMR**  H-4 of pyrimidine  **^13^C NMR**  C-4 of pyrimidine |
| 118.34  141.22 - | 6.28 (d) | 16.35 – 150.28 | 6.64 | 108.79 – 149.48 | 6.84 | 111.05 – 136.15 | 6.54 | **^1^H NMR**  H-5 of pyrimidine  **^13^C NMR**  C-aryl |
| 150.11, 153.34 | 6.57 (d) | 138.31, 156.19 | 6.40 | 136.85, 153.74 | 6.35 | 163.11, 127.75 | 6.34 | **^1^H NMR**  3H of pyrrole **^13^C NMR**  2C=N |
| --- | 7.11 (dd) | --- | 6.51 | --- | 6.47 | --- | 6.56 | **^1^H NMR**  H-4 of pyrrole |
| --- | 7.25 (d)) | --- | 7.00 | --- | 6.94 | --- | 6.87 | **^1^H NMR**  H- 5 of pyrrole |
| --- | 7.38 – 8.26 | --- | 7.14 – 8.60 | --- | 7.31-8.58 | --- | 7.31 – 7.95 | **^1^H NMR**  Hs of thiophene and phenyl |
| **L4** | | | | | | | | |
| 25.54 | 1.89 (S) | 18.00 | 2.72, 3.11 | 20.60 | 2.68, 2.69, 3.07 | 20.42 | 2.68, 2.69, 3.07 | **^1^H NMR**  3H, CH3 of pyrazol  **^13^C NMR**  CH_3_ |
| 49.34 | 2.89 (S) | 43.12 | 4.61, 4.82 | 46.04 | 4.65, 4.87 | 45.84 | 4.65, 4.87 | **^1^H NMR**  CH2 of pyrazol  **^13^C NMR**  NCH_3_ |
| 69.75 | 3.63 (S) | 60.17 | 4.22, 4.34, 4.42 | 63.85 | 4.29, 4.39, 4.47 | 64.85 | 4.29, 4.39, 4.47 | **^1^H NMR**  NCH3  **^13^C NMR**  C-4 of pyrimidine |
| 119.19 -145.77 | 5.94 (d) | 108.06 – 155.30 | 5.64 | 110.87 – 154.88 | 5.62 | 111.76 – 152.67 | 5.97 | **^1^H NMR**  H-4 of pyrimidine  **^13^C NMR**  Aryl C |
| 158.73  ,160.22 | 6.44 (d) | 145.72, 152.59 | 6.67 | 144.55, 150.15 | 6.93 | 143.75, 150.10 | 6.93 | **^1^H NMR**  H-5 of pyrimidine  **^13^C NMR**  2 C=N |
| 177.04 | 6.73 (d)) | 195.65 | 6.37 | 193.13 | 6.33 | 192.08 | 6.33 | **^1^H NMR**  H-3 of pyrrole  **^13^C NMR**  C=O |
| --- | 7.31 (dd) |  | 6.53 |  | 6.48 |  | 6.48 | **^1^H NMR**  H-4 of pyrrole |
| --- | 7.51 (d) |  | 7.09 |  | 7.04 |  | 7.04 | **^1^H NMR**  H-5 of pyrrole |
| --- | 7.48 (d) |  | 7.49 |  | 7.47 |  | 7.47 | **^1^H NMR**  H-3 of thiophene |
| --- | 7.82 (dd) |  | 7.60 |  | 7.55 |  | 7.55 | **^1^H NMR**  H-4 of thiophene |
| --- | 8.17 (d) |  | 7.66 |  | 7.58 |  | 7.58 | **^1^H NMR**  H-5 of thiophene |
| --- | 9.38 (S, br), |  | 5.92 |  | 5.97 |  | 5.97 | **^1^H NMR**  NH of pyrimidine |
| **L5** | | | | | | | | |
| --- | 3.37 (S) |  | 4.21, 4.28, 4.38 |  | 4.28, 4.34, 4.43 |  | 3.65, 3.91 | **^1^H NMR**  NCH3 |
| --- | 5.38 (d) |  | 5.39 |  | 5.39 |  | 4.86 | **^1^H NMR**  H-4 of pyrimidine |
| --- | 6.23 (d) |  | 7.54 |  | 7.84 |  | 6.65 | **^1^H NMR**  H-5 of pyrimidine |
| --- | 6.81 (d) |  | 6.39 |  | 6.35 |  | 6.26 | **^1^H NMR**  H-3 of pyrrole |
| --- | 7.32 (dd) |  | 6.73 |  | 6.68 |  | 6.75 | **^1^H NMR**  H-4 of pyrrole |
| --- | 7.53 (d) |  | 7.04 |  | 6.98 |  | 6.85 | **^1^H NMR**  H-5 of pyrrole |
| --- | 7.89 (d) |  | 7.49 |  | 7.34 |  | 7.25 | **^1^H NMR**  H-3 of thiophen |
| --- | 7.99 (dd) |  | 7.66 |  | 7.64 |  | 7.46 | **^1^H NMR**  H-4 of thiophene |
| --- | 8.11 (d) |  | 7.72 |  | 7.68 |  | 7.67 | **^1^H NMR**  H-5 of thiophene |
| --- | 8.42 (S) |  | 8.18 |  | 7.96 |  | 7.46 | **^1^H NMR**  H-3 of triazole |
| --- | 12.83 (S. br) |  | 9.58 |  | 9.37 |  | 7.95 | **^1^H NMR**  NH of triazole |

**Table S4.** Values of the mulliken charges, Fukui function of L1

|  | N | N-1 | N+1 | 𝑓𝑘 ^–^ | 𝑓𝑘 ^+^ | F^0^ |
| --- | --- | --- | --- | --- | --- | --- |
| C1 | 0.010 | 0.036 | -0.003 | -0.025 | -0.013 | -0.039 |
| C2 | -0.205 | -0.265 | -0.119 | **0.060** | **0.086** | **0.146** |
| C3 | 0.347 | 0.291 | 0.382 | **0.057** | 0.034 | **0.091** |
| N1 | -0.499 | -0.519 | -0.492 | 0.020 | 0.007 | 0.027 |
| N2 | -0.587 | -0.581 | -0.571 | -0.006 | 0.016 | 0.010 |
| N3 | -0.463 | -0.464 | -0.472 | 0.001 | -0.009 | -0.008 |
| C4 | 0.386 | 0.322 | 0.381 | **0.065** | -0.005 | 0.059 |
| C5 | -0.082 | -0.122 | -0.068 | 0.040 | 0.015 | 0.055 |
| C6 | -0.067 | -0.076 | -0.059 | 0.009 | 0.008 | 0.017 |
| C7 | -0.304 | -0.335 | -0.292 | 0.031 | 0.012 | 0.044 |
| C8 | -0.129 | -0.140 | -0.136 | 0.011 | -0.006 | 0.004 |
| C9 | 0.079 | 0.048 | 0.171 | 0.031 | **0.092** | **0.123** |
| C10 | 0.216 | 0.206 | 0.283 | 0.010 | **0.067** | 0.077 |
| C11 | -0.172 | -0.200 | -0.122 | 0.028 | **0.051** | 0.079 |
| C12 | -0.168 | -0.176 | -0.135 | 0.007 | 0.033 | 0.040 |
| C13 | -0.195 | -0.185 | -0.211 | -0.011 | -0.015 | -0.026 |
| S1 | -0.449 | -0.645 | -0.295 | **0.196** | **0.154** | **0.350** |
| S2 | 0.237 | 0.145 | 0.273 | **0.091** | 0.036 | **0.127** |

**Table S5.** Values of the mulliken charges, Fukui function of L2

|  | N | N-1 | N+1 | 𝑓𝑘 ^–^ | 𝑓𝑘 ^+^ | F0 |
| --- | --- | --- | --- | --- | --- | --- |
| C1 | -0.019 | -0.009 | -0.040 | -0.010 | -0.021 | -0.031 |
| C2 | -0.182 | -0.190 | -0.166 | 0.008 | 0.016 | 0.024 |
| C3 | 0.288 | 0.258 | 0.299 | 0.030 | 0.011 | 0.041 |
| N1 | -0.492 | -0.486 | -0.441 | -0.007 | **0.051** | 0.044 |
| N2 | -0.546 | -0.551 | -0.548 | 0.005 | -0.001 | 0.004 |
| N3 | -0.469 | -0.470 | -0.470 | 0.001 | -0.001 | 0.000 |
| C4 | 0.010 | 0.020 | -0.018 | -0.010 | -0.028 | -0.037 |
| C5 | -0.085 | -0.128 | -0.073 | **0.043** | 0.012 | 0.055 |
| C6 | -0.069 | -0.093 | -0.065 | 0.024 | 0.004 | 0.028 |
| C7 | -0.308 | -0.366 | -0.300 | **0.057** | 0.008 | **0.066** |
| C8 | -0.123 | -0.162 | -0.132 | 0.039 | -0.009 | 0.030 |
| C9 | 0.072 | 0.053 | 0.084 | 0.019 | 0.012 | 0.031 |
| C10 | 0.211 | 0.205 | 0.217 | 0.007 | 0.005 | 0.012 |
| C11 | -0.174 | -0.192 | -0.168 | 0.018 | 0.006 | 0.025 |
| C12 | -0.171 | -0.175 | -0.167 | 0.004 | 0.004 | 0.008 |
| C13 | -0.193 | -0.187 | -0.197 | -0.006 | -0.005 | -0.010 |
| S1 | 0.107 | -0.002 | 0.391 | **0.109** | **0.284** | **0.394** |
| S2 | 0.224 | 0.071 | 0.248 | **0.153** | 0.024 | **0.177** |
| C14 | -0.242 | -0.240 | -0.160 | -0.002 | **0.082** | **0.080** |
| C15 | 0.630 | 0.609 | 0.716 | 0.021 | 0.086 | **0.107** |
| C16 | -0.039 | -0.037 | -0.071 | -0.002 | -0.033 | -0.034 |
| O1 | -0.466 | -0.473 | -0.408 | 0.007 | **0.058** | **0.065** |
| N4 | -0.270 | -0.283 | -0.238 | 0.012 | 0.033 | 0.045 |
| C17 | -0.125 | -0.130 | -0.116 | 0.005 | 0.009 | 0.014 |
| C18 | -0.101 | -0.115 | -0.097 | 0.014 | 0.004 | 0.019 |
| C19 | -0.094 | -0.104 | -0.086 | 0.010 | 0.008 | 0.018 |
| C20 | -0.103 | -0.106 | -0.098 | 0.003 | 0.004 | 0.008 |
| C21 | -0.126 | -0.144 | -0.123 | 0.018 | 0.003 | 0.021 |
| C22 | 0.096 | 0.095 | 0.097 | 0.001 | 0.002 | 0.003 |

**Table S6**. Values of the mulliken charges, Fukui function of L3

|  | N | N-1 | N+1 | 𝑓𝑘 ^–^ | 𝑓𝑘 ^+^ | F^0^ |
| --- | --- | --- | --- | --- | --- | --- |
| C1 | -0.028 | -0.008 | -0.048 | -0.020 | -0.020 | -0.040 |
| C2 | -0.174 | -0.177 | -0.073 | 0.003 | **0.101** | **0.104** |
| C3 | 0.286 | 0.274 | 0.321 | 0.012 | 0.035 | 0.047 |
| N1 | -0.454 | -0.455 | -0.449 | 0.001 | 0.005 | 0.006 |
| N2 | -0.551 | -0.552 | -0.518 | 0.001 | 0.033 | 0.034 |
| N3 | -0.469 | -0.469 | -0.479 | 0.000 | -0.010 | -0.010 |
| C4 | 0.003 | 0.013 | -0.018 | -0.010 | -0.021 | -0.032 |
| C5 | -0.089 | -0.105 | -0.076 | 0.015 | 0.014 | 0.029 |
| C6 | -0.070 | -0.079 | -0.062 | 0.010 | 0.008 | 0.018 |
| C7 | -0.307 | -0.330 | -0.295 | 0.024 | 0.012 | 0.036 |
| C8 | -0.117 | -0.137 | -0.123 | 0.020 | -0.006 | 0.014 |
| C9 | 0.072 | 0.066 | 0.166 | 0.006 | **0.094** | **0.100** |
| C10 | 0.212 | 0.211 | 0.277 | 0.001 | **0.065** | **0.066** |
| C11 | -0.174 | -0.179 | -0.120 | 0.005 | 0.055 | 0.059 |
| C12 | -0.171 | -0.173 | -0.139 | 0.002 | 0.032 | 0.034 |
| C13 | -0.191 | -0.189 | -0.209 | -0.003 | -0.018 | -0.020 |
| S1 | 0.229 | 0.141 | 0.286 | **0.088** | 0.056 | **0.145** |
| S2 | 0.226 | 0.158 | 0.263 | **0.067** | 0.037 | **0.104** |
| N4 | -0.586 | -0.644 | -0.567 | 0.058 | 0.019 | **0.077** |
| C14 | 0.425 | 0.363 | 0.432 | 0.062 | 0.007 | 0.069 |
| C15 | -0.099 | -0.134 | -0.096 | 0.035 | 0.003 | 0.038 |
| C16 | -0.104 | -0.130 | -0.102 | 0.026 | 0.002 | 0.028 |
| C17 | -0.089 | -0.159 | -0.086 | **0.070** | 0.003 | **0.073** |
| C18 | -0.103 | -0.121 | -0.100 | 0.018 | 0.003 | 0.021 |
| C19 | -0.111 | -0.152 | -0.114 | 0.041 | -0.003 | 0.038 |
| C20 | 0.051 | 0.021 | 0.046 | 0.030 | -0.005 | 0.025 |

**Table S7.** Values of the mulliken charges, Fukui function of L4

|  | N | N-1 | N+1 | 𝑓𝑘 ^–^ | 𝑓𝑘 ^+^ | F0 |
| --- | --- | --- | --- | --- | --- | --- |
| C1 | 0.009 | 0.017 | -0.014 | -0.008 | -0.023 | -0.032 |
| C2 | -0.204 | -0.232 | -0.092 | 0.028 | **0.112** | **0.140** |
| C3 | 0.291 | 0.284 | 0.320 | 0.007 | 0.028 | 0.035 |
| N1 | -0.569 | -0.570 | -0.550 | 0.001 | 0.020 | 0.020 |
| N2 | -0.642 | -0.664 | -0.601 | 0.021 | 0.042 | 0.063 |
| N3 | -0.475 | -0.474 | -0.484 | -0.001 | -0.009 | -0.010 |
| C4 | 0.801 | 0.744 | 0.848 | **0.057** | 0.047 | **0.104** |
| C5 | -0.087 | -0.094 | -0.072 | 0.007 | 0.015 | 0.022 |
| C6 | -0.068 | -0.070 | -0.062 | 0.002 | 0.006 | 0.008 |
| C7 | -0.307 | -0.311 | -0.298 | 0.004 | 0.009 | 0.013 |
| C8 | -0.129 | -0.124 | -0.139 | -0.005 | -0.009 | -0.015 |
| C9 | 0.069 | 0.062 | 0.149 | 0.007 | **0.080** | 0.087 |
| C10 | 0.218 | 0.216 | 0.272 | 0.003 | 0.054 | 0.056 |
| C11 | -0.161 | -0.165 | -0.108 | 0.004 | 0.053 | 0.057 |
| C12 | -0.176 | -0.178 | -0.150 | 0.002 | 0.026 | 0.029 |
| C13 | -0.192 | -0.188 | -0.211 | -0.004 | -0.018 | -0.023 |
| S1 | 0.225 | 0.214 | 0.261 | 0.012 | 0.035 | 0.047 |
| C14 | 0.286 | 0.198 | 0.310 | **0.087** | 0.024 | **0.112** |
| C15 | 0.323 | 0.201 | 0.333 | 0.122 | 0.010 | **0.132** |
| C16 | -0.066 | -0.050 | -0.069 | -0.016 | -0.003 | -0.019 |
| C17 | -0.370 | -0.370 | -0.371 | 0.000 | -0.001 | -0.001 |
| N4 | -0.306 | -0.438 | -0.302 | 0.132 | 0.004 | **0.137** |
| N5 | -0.394 | -0.386 | -0.393 | -0.008 | 0.000 | -0.007 |
| O1 | -0.499 | -0.638 | -0.479 | 0.139 | 0.020 | **0.159** |

**Table S8.** Values of the mulliken charges, Fukui function of L5

|  | N | N-1 | N+1 | 𝑓𝑘 ^–^ | 𝑓𝑘 ^+^ | F0 |
| --- | --- | --- | --- | --- | --- | --- |
| C1 | 0.040 | 0.049 | 0.015 | -0.009 | -0.024 | -0.033 |
| C2 | -0.232 | -0.259 | -0.110 | 0.027 | 0.123 | **0.149** |
| C3 | 0.288 | 0.241 | 0.313 | 0.047 | 0.025 | 0.072 |
| N1 | -0.529 | -0.515 | -0.521 | -0.014 | 0.008 | -0.006 |
| N2 | -0.658 | -0.669 | -0.582 | 0.012 | 0.075 | 0.087 |
| N3 | -0.476 | -0.477 | -0.482 | 0.001 | -0.007 | -0.005 |
| C4 | 0.818 | 0.759 | 0.873 | 0.059 | 0.055 | **0.114** |
| C5 | -0.084 | -0.139 | -0.069 | 0.055 | 0.015 | 0.070 |
| C6 | -0.068 | -0.091 | -0.061 | 0.024 | 0.006 | 0.030 |
| C7 | -0.304 | -0.365 | -0.294 | 0.061 | 0.010 | 0.070 |
| C8 | -0.139 | -0.179 | -0.152 | 0.039 | -0.013 | 0.027 |
| C9 | 0.068 | 0.040 | 0.129 | 0.029 | 0.061 | **0.090** |
| C10 | 0.207 | 0.200 | 0.244 | 0.007 | 0.037 | 0.044 |
| C11 | -0.160 | -0.185 | -0.118 | 0.026 | 0.042 | 0.067 |
| C12 | -0.176 | -0.181 | -0.155 | 0.005 | 0.021 | 0.026 |
| C13 | -0.193 | -0.183 | -0.211 | -0.009 | -0.018 | -0.027 |
| S1 | 0.234 | 0.058 | 0.275 | 0.176 | 0.041 | **0.217** |
| N4 | -0.461 | -0.476 | -0.429 | 0.015 | 0.031 | 0.047 |
| N5 | -0.309 | -0.334 | -0.271 | 0.026 | 0.038 | 0.064 |
| C14 | 0.306 | 0.290 | 0.341 | 0.016 | 0.035 | 0.051 |


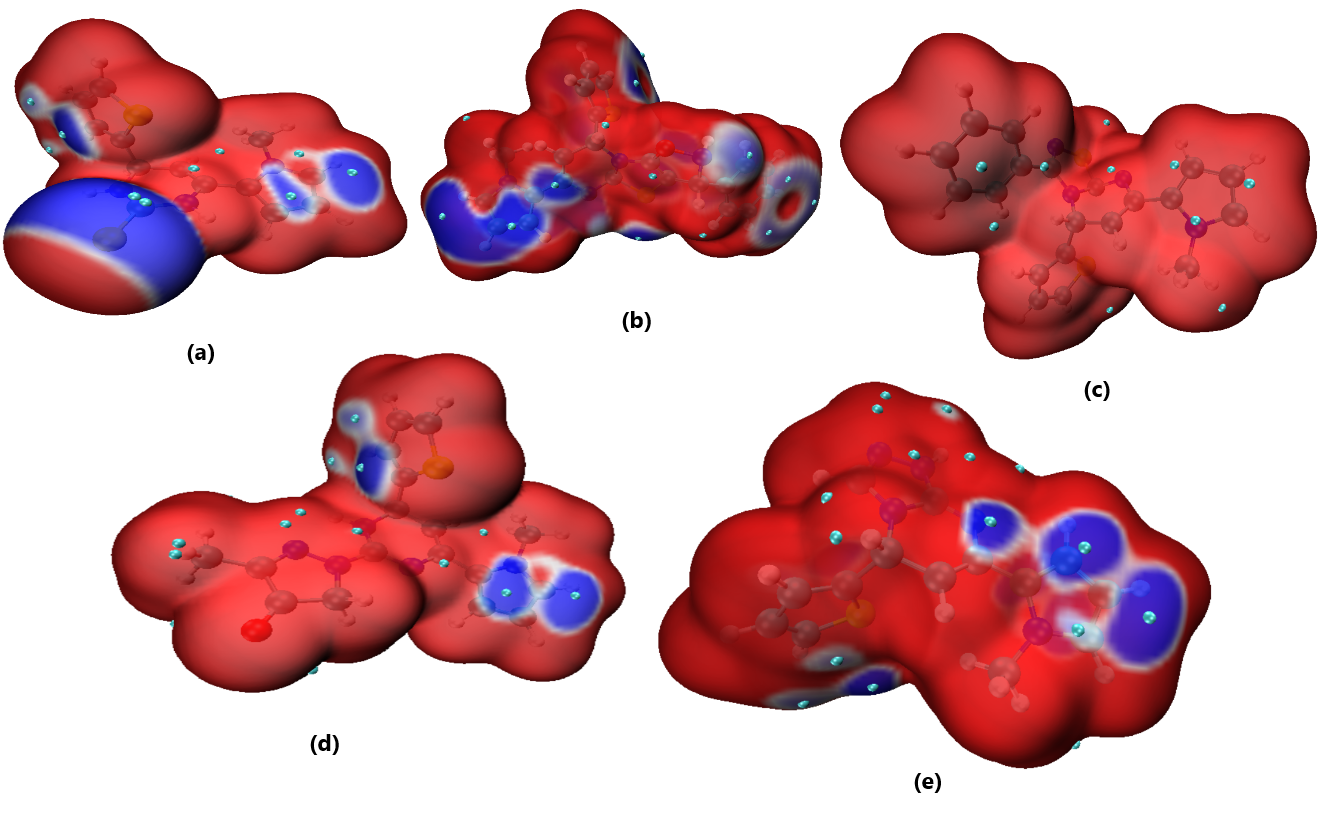


**Figure S1.** ALIE of the heterocyclic systems (a) L1, (b) L2, (c) L3, (d) L4, (e) L5
